# L-Cysteine Increases the Transformation Efficiency of Chinese Cabbage (*Brassica rapa* ssp. *pekinensis*)

**DOI:** 10.3389/fpls.2021.767140

**Published:** 2021-10-26

**Authors:** Ganeshan Sivanandhan, Jiae Moon, Chaemin Sung, Solhee Bae, Zhi Hong Yang, So Young Jeong, Su Ryun Choi, Sang-Gyu Kim, Yong Pyo Lim

**Affiliations:** ^1^Molecular Genetics and Genomics Laboratory, Department of Horticulture, College of Agriculture and Life Science, Chungnam National University, Daejeon, South Korea; ^2^Department of Biological Sciences, KAIST, Daejeon, South Korea

**Keywords:** *Agrobacterium*, ascorbic acid, Chinese cabbage, *Brassica rapa*, L-Cysteine, silver nitrate, transgenic regeneration, optimization of factors

## Abstract

Successful *Agrobacterium*-mediated transformations of Chinese cabbage have been limited owing to the plant’s recalcitrant nature, genomic background and explant necrosis upon infection, which hinders the transfer of T-DNA region into the Chinese cabbage. Consequently, in the current experiment, a stable *Agrobacterium tumefaciens-*mediated transformation method for Chinese cabbage cv. Kenshin established by employing important anti-oxidants in the co-cultivation and subsequent regeneration media. Four-day-old *in vitro* derived cotyledon explants were infected with *A. tumefaciens* strain GV3101 harboring the vector pCAMIBA1303. Cotyledon explants exposed to an *Agrobacterium* suspension (OD_600_ of approximately 0.6) for 10 min and then incubated for 3 days co-cultivation in Murashige and Skoog medium containing an L-cysteine + AgNO_3_ combination exhibited the highest β-glucuronidase (GUS) expression (94%) and explant regeneration efficiency (76%). After 3 days, the cotyledon explants were subjected to three selection cycles with gradually increasing hygromycin B concentrations (10 to 12 mg/L). The incorporation and expression of *hptII* in T_0_ transformed plants were verified by polymerase chain reaction and Southern blot analyses. These transgenic plants (T_0_) were fertile and morphologically normal. Using the present protocol, a successful transformation efficiency of 14% was achieved, and this protocol can be applied for genome editing and functional studies to improve Chinese cabbage traits.

## Introduction

Chinese cabbage (*Brassica rapa* ssp. *pekinensis*; *Brassicaceae*) is an important leafy vegetable crop that is cultivated worldwide. It has abundant healthful properties and is rich in phytochemicals, such as phenolics, carotenoids, flavonoids, glucosinolates, and anthocyanins. It reduces the effects of some diseases, such as cancer, heart diseases, diabetes, osteoporosis and high blood pressure.^1^ China, India and Russia are the chief producers of Chinese cabbage. The production of cabbage and other *Brassicas* in South Korea during 2018--2019 was 2.57 million tons, making South Korea the fifth largest producer in the world.^2^ During 2016–2017, South Korea imported 13.7 thousand tons of cabbage and related vegetables, having a US $5.2 million import value (see text footnote 2). Thus, the demand for Chinese cabbage is increasing annually, but the present annual production does not meet the demand (see text footnote 2). Consequently, increasing the production of this crop is essential. Increasing the cultivation land for Chinese cabbage is one solution, but it does not increase the production rate. Thus, determining the best alternative solution to increased land usage is a potentially important strategy. The major constraints on Chinese cabbage production include several biotic and abiotic factor-related issues, such as bacterial soft rot disease caused by *Pectobacterium carotovorum*, which results in significant losses in crop productivity and quality (Kim et al., 2014). Although, conventional plant breeding methods have significantly improved Chinese cabbage, certain limitations, such as a complex genome, susceptibility to different stresses and long selection regimes, have limited its development (Velasco et al., 2017).

Under these circumstances, plant tissue culturing and transformation are vital for the development of elite Chinese cabbage plants. *Agrobacterium*-mediated genetic transformation is an important method that has been used to develop several transgenic plants in short time spans. However, the *in vitro* regeneration and transformation of *B. rapa* are difficult owing to the plant’s recalcitrant nature (Burnett et al., 1994). Despite this recalcitrance, genetic development through *Agrobacterium*-mediated transformation is a powerful way to improve *B. rapa* traits. There are limited available reports on the *Agrobacterium*-mediated transformation of Chinese cabbage (Jun et al., 1995; Zhang et al., 2000; Lee et al., 2004; Ku et al., 2006; Min et al., 2007; Konagaya et al., 2013; Baskar et al., 2016; Liu et al., 2018; Li et al., 2021). Jun et al. (1995) reported 3% of transformation efficiency from cotyledon explants of Chinese cabbage (*B. campestris* L. ssp. *pekinensis*). Zhang et al. (2000) obtained transformation frequencies in ranges of 1.6–2.7% in three cultivars of cotyledon explants of Chinese cabbage (*B. campestris* L. ssp. *pekinensis*). Lee et al. (2004) generated transgenic Chinese cabbage (*B. rapa* L. ssp. *pekinensis*) from hypocotyl explants with transformation efficiencies ranging from 2.89 to 5%. Ku et al. (2006) reported 83.3% of transformation efficiency in *B. rapa* based on Polymerase chain reaction (PCR) confirmation. Transformation efficiencies were 1.4 to 3% in hypocotyl explants of Chinese cabbage (*B. rapa*) using mannose as a selection agent (Min et al., 2007). Transformation efficiency was 1.2% in hypocotyl explants of Chinese cabbage (*B. rapa* L. ssp. *pekinensis*) when bispyribac sodium used as a selection agent (Konagaya et al., 2013). Baskar et al. (2016) obtained 15% efficiency of transformation using hypocotyl explants of Chinese cabbage (*B. rapa* L. ssp. *pekinensis*) based on PCR confirmation and used hygromycin as a selection agent. Liu et al. (2018) achieved 0.6 to 1.2% of transformation efficiency using heading leaves of Chinese cabbage (*B. rapa* L. ssp. *pekinensis*) and used kanamycin as a selection agent. Li et al. (2021) attained 10.83% of transformation efficiency in cotyledon explants of Chinese cabbage (*B. rapa* L. ssp. *pekinensis*) using hygromycin as a selection agent.

Although earlier reports showed successful *Agrobacterium*-mediated transformations of Chinese cabbage, several limitations, such as low transformation efficiencies, limited shoot regeneration, unstable integration, enzymatic browning, wound-induced phenolic compound secretion, insufficient scorable gene analyses and lack of repeatability, have hindered the development of Chinese cabbage harboring favorable traits (Li et al., 2018). Because of unstable genetic transformation, many beneficial traits (for instance increased yield and drought resistant traits) that have been enhanced in *Arabidopsis*, rice and wheat have not been improved in Chinese cabbage.

Tissue browning and necrosis are common problems in *Agrobacterium*-mediated transformation in plants (Dan, 2008). The explants when infected with *Agrobacterium* have attained defense responses followed by formation of the protective coating around the wound. As a result, tissue necrosis and browning would result. Recently, antioxidants like L-cysteine, ascorbic acid have used in growing media in order to control the browning issues upon *Agrobacterium* infection (Dan, 2008). When the antioxidants were used in the growing media, the tissue necrosis and browning can significantly reduce phenolic exudation in infected explants, and thereby increase the regeneration efficiency followed by improving the transformation efficiency. The addition of L-cysteine, ascorbic acid and silver nitrate in co-cultivation medium has also been revealed to considerably recover transformation efficiency in rice (Enríquez-Obregón et al., 1999) and Kodo millet (Bhatt et al., 2021). It has also been confirmed that L-cysteine improves the *Agrobacterium*-mediated transformation in soybean by reducing oxidative stress (Olhoft et al., 2001). The viability of explants followed by recovery of transgenic plants were improved when L-cysteine added in the co-cultivation and selection media in finger millet transformation (Antony Ceasar and Ignacimuthu, 2011). Application of the media that containing L-cysteine, dithiothreitol and sodium thiosulphate showed higher transformation efficiency in *Withania somnifera* transformation (Sivanandhan et al., 2015). Liu et al. (2017) reported that cysteine has significantly improved the transformation efficiency in maize and confirmed oxidative reduction related genes were upregulated in cysteine treated plant cells.

In Chinese cabbage transformations, enzymatic browning and tissue necrosis are major issues that lead to low levels of T-DNA delivery and competent cell regeneration from explants (Li et al., 2018). Thus, we have attempted to enhance the transformation efficiency of Chinese cabbage for the first time through the mitigation of tissue browning by adding L-cysteine and ascorbic acid; thereby triggering T-DNA delivery into cotyledon explants.

## Materials and Methods

### Plant Material and Regeneration

Four-day-old cotyledon explants of *B. rapa* cv. Kenshin were used for the transformation study. Seed germination and regeneration protocols were published previously (Sivanandhan et al., 2019).

### Sensitivity of Cotyledon Explants to Hygromycin B

The vector used in the present study harbored *hygromycin phosphotransferase* (*hpt*II) as a plant selection marker. The best hygromycin B concentration was determined for the selection of transgenic shoots by *in vitro* culturing cotyledon explants on shoot-induction medium supplemented with several hygromycin B concentrations (0, 2, 4, 6, 8, 10 mg/L) along with optimum concentrations of benzyl adenine (BA) at 5 mg/L, 1-naphthaleneacetic acid (NAA) at 0.5 mg/L and silver nitrate (AgNO_3_) at 4 mg/L. Plant tissue necrosis was recorded after 12 weeks of shoot production. An appropriate control without hygromycin B addition was maintained for explants. The cultures were passed to the similar medium containing same levels of antibiotics every 2 weeks for 12 weeks, and then, responses of shoot regeneration were determined. The cultures were maintained at 25 ± 2°C under a 16-h photoperiod (50 μmol m^–2^ s^–*l*^).

### *Agrobacterium tumefaciens* Strain and Preparation for Infection

In our transformation study, *A. tumefaciens* strain GV3101, harboring the binary vector pCAMBIA1303, which carries *hygromycin phosphotransferase* (*hptII)* as a selection marker and β*-glucuronidase A*:*green fluorescent protein 5* (*gusA-mgfp5*) as a fusion gene scorable marker, was used. These genes were under the control of the CaMV35S promoter. The *neomycin phosphotransferase* (*npt* II) gene (kanamycin resistant) was in the vector backbone and allows bacterial selection. The preparation and maintenance of the *A. tumefaciens* culture were achieved as defined earlier (Sivanandhan et al., 2016).

### Chinese Cabbage Transformation

The petiole regions of the cotyledon explants were wounded softly by pricking 2–3 times with a sterile needle, and the wounded explants were moved to Petri plates comprising 30 ml of *Agrobacterium* culture with 5 mg/L acetosyringone at an OD_600_ of approximately 0.6–0.7, with the explants immersed in the *Agrobacterium* suspension culture. The Petri plates comprising the explants were rotated at 30 rpm for 10 min in a bacterial suspension to promote an efficient infection rate. The *Agrobacterium* culture-treated explants were blotted dry on filter paper and then transferred to co-cultivation medium containing optimum concentrations of BA (5 mg/L), and NAA (0.5 mg/L) [control] for a 3-day incubation in darkness. In a separate experiment, the infected cotyledons were cultured on co-cultivation medium augmented with various concentrations of L-cysteine (0, 200, 400, 600, 800, 1000 mg/L), ascorbic acid (0, 10, 20, 30, 40, 50 mg/L) and AgNO_3_ (0, 1, 2, 3, 4, 5 mg/L) in Murashige and Skoog (MS; Murashige and Skoog, 1962) medium that contained BA (5 mg/L) and NAA (0.5 mg/L) to increase the transformation efficiency of Chinese cabbage. After optimizing these components, the components at their ideal concentrations were combined to increase the shoot regeneration rate from infected explants. After treatment, the explants were rinsed with sterile distilled water (approximately three times) and then in hormonal MS liquid medium (shoot-induction medium) containing the optimal concentration of L-cysteine, ascorbic acid or AgNO_3_ and 500 mg/L timentin, or in each component’s optimal concentration combined with timentin. For the control culture, the *Agrobacterium* washes were performed using hormonal MS liquid medium with timentin alone. The cotyledon explants were desiccated and subjected to three selection cycles to improve stable transgenic shoots. The first cycle was undertaken on MS shoot-selection medium supplemented with optimal concentrations of BA (5 mg/L), NAA (0.5 mg/L), L-cysteine + AgNO_3_ (800 mg/L + 4 mg/L), timentin (500 mg/L) and hygromycin B (10 mg/L) for 4 weeks with subculturing at weekly intervals. The green shoots regenerated from cotyledons after the first selection cycle were again subjected to a second selection cycle on MS shoot-selection medium containing BA, NAA, L-cysteine + AgNO_3_, timentin and 11 mg/L hygromycin B for 4 weeks with subculturing at weekly intervals. Likewise, a third cycle was undertaken on MS shoot-selection medium supplemented with BA, NAA, L-cysteine + AgNO_3_, timentin and 12 mg/L hygromycin B for 4 weeks with subculturing at weekly intervals. After 12 weeks of selection cycles having gradually increasing hygromycin B concentrations, the shoots that reached 4–5 cm heights and survived on shoot-selection medium were removed from the shoot pad and transferred to half-strength MS medium supplemented with timentin (500 mg/L) and 12 mg/L hygromycin B to undergo root induction for 4 weeks.

### Establishment of Transgenic Plants

Hardening was performed as per our previously published method (Sivanandhan et al., 2019). After 7 weeks in a controlled growth room, all the plants (T_0_) were moved to large pots containing autoclaved perlite and transplanted in a glasshouse.

### GUS Expression

The β-glucuronidase (GUS) test was achieved to confirm the presence of the *gusA* gene in the transgenic tissues (Jefferson et al., 1987). After infection with *Agrobacterium*, explants were co-cultivated on selection medium for 10 days. Then, the explants were washed several times with MS medium supplemented with 500 mg/L timentin, followed by sterile distilled water and 100% methanol washes. The control and transgenic plants and their parts were incubated overnight at 37°C. The pigments were separated from the explants using 100% (v/v) methanol overnight.

### PCR Confirmation and Copy Number Estimation by Southern Blot Analysis

DNA was isolated from four randomly selected hygromycin B-resistant plants, as well as from control plants, using a DNA isolation kit (Sigma-Aldrich, United States) to confirm the existence of the *hptII* gene in the transgenic plants using a *hptII* primer pair and a PCR analysis (C1000^TM^ Thermal Cycler, Bio-Rad Laboratories, United States). The *hptII* gene fragment (407 bp) was amplified using the FP 5′-GATGTTGGCGACCTCGTATT-3′ and RP 5′-GTGTCACGTTGCAAGACCTG-3′ primer pair. Polymerase chain reaction (PCR) amplifications were performed with an initial DNA denaturation at 94°C for 4 min, followed by 30 cycles of 94°C for 1 min, 58°C for 1 min and 72°C for 1 min, and then a final extension at 72°C for 7 min. Non-transformed plants and plasmid DNA from pCAMBIA1303 genomic DNA were used as negative and positive controls, respectively. The PCR products were examined in 1% (w/v) agarose gels.

The total isolated genomic DNA (15 μg) samples from transgenic plants and non-transformed control plants were digested for 8 h using *Eco*RI (Thermo Scientific, Pittsburgh, PA, United States) and separated using 1% (w/v) agarose gel electrophoresis. Capillary transfer was then performed to nylon membranes (Hybond N^+^, Amersham Biosciences, England) as per the method of Sambrook and Russell (2001). The amplified *hptII* PCR product was purified and labeled with a DIG High Prime probe for synthesis (Roche, United States). Remaining steps, such as pre-hybridization, post-hybridization, detection, washing and exposure, were in accordance with the DIG High Prime DNA Labeling and Detection Starter Kit II manual.

### Statistical Analysis

A completely randomized design was used for the present study. The tests were repeated three times with three replicates. Data are presented as means. The mean separations were performed using Duncan’s multiple range test, and significance was determined at the 5% level (SPSS 17.5; IBM, Armonk, New York, United States).

## Results

### Sensitivity of Cotyledon Explants to Hygromycin B

**FIGURE 1:**
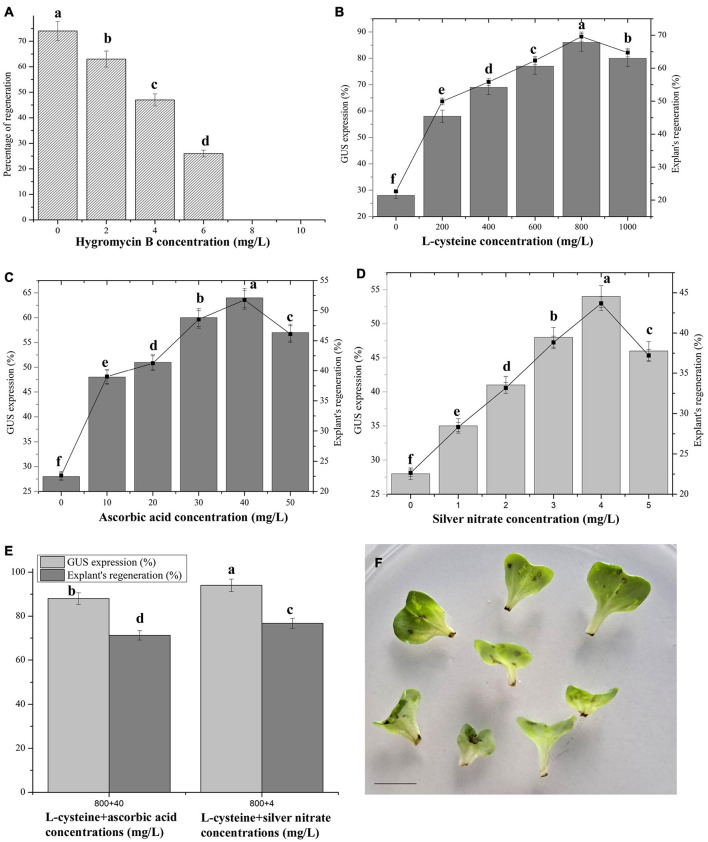
*Agrobacterium*-mediated transformation in Chinese cabbage. **(A)** Sensitivity of cotyledon explants to hygromycin B. Effect of different antioxidants such as L-cysteine **(B),** ascorbic acid **(C),** silver nitrate **(D),** combined effects of best concentrations of L-cysteine + ascorbic acid, and L-cysteine + silver nitrate on GUS expression, and explant’s regeneration efficiencies **(E).** **(F)** Necrosis of petiole region of explants after *Agrobacterium* infection. Values represent the mean ± standard error of three replicates. One hundred twenty-eight cotyledon explants were infected for each treatment and the experiments were repeated three times. Means with the same letter above bars are not significantly different at 5% level according to Duncan’s multiple range test.

The cotyledon explants were extremely susceptible to different hygromycin B concentrations, and the shoot induction capacity dramatically decreased in cotyledon explants as the hygromycin B concentration increased (Figure 1A). A slight reduction in the concentration resulted in a weak toxicity to the growing explants, whereas higher hygromycin B concentrations resulted in decreases in explant growth and development (Figure 1A). Single shoot/explant in 2 and 4 mg/L hygromycin B concentrations showed 63% and 47% shoot-induction responses, respectively. However, the cotyledons were pale green (26%) in 6 mg/L hygromycin B and no regeneration occurred. All the explants died in 8 and 10 mg/L hygromycin B concentrations. On the basis of this study, 10 mg/L hygromycin B was selected as an ideal concentration for the selection of transgenic shoots.

### L-Cysteine’s Effects on GUS Expression and Regeneration Efficiency

Supplementation with L-cysteine significantly elevated the number of GUS positive responses, as well as the percentage of explant regeneration, in Chinese cabbage compared with the control (Figure 1B). All the tested L-cysteine concentrations had positive effects on GUS expression compared with the control. L-Cysteine at 800 mg/L in co-the cultivation medium resulted in an 86% GUS expression and 69.57% shoot regeneration response from cotyledon explants (Figure 1B). The percentages of GUS expression and explant regeneration were significantly increased, each being threefold higher than in the control cultures. The addition of L-cysteine did not affect meristematic cell formation or the following dedifferentiation even when supplemented at 800 mg/L in the selection medium, whereas at higher concentrations, explant growth deteriorated and they died (data not shown).

### Ascorbic Acid’s Effects on GUS Expression and Regeneration Efficiency

Among them, 40 mg/L in the co-cultivation medium exhibited maximum GUS expression (64%) and percentage of explant regeneration (51.77%) levels (Figure 1C). The results obtained at 40 mg/L were 2.28- and 2.28-fold greater than the control for GUS expression and percentage of explant regeneration, respectively. Low concentrations (10–30 mg/L) or a high concentration (50 mg/L) of ascorbic acid did not produce significant results, but they were greater than those of the control cultures. The high concentration led to suppressed explant growth.

### AgNO_3_’s Effects on GUS Expression and Regeneration Efficiency

Among the various tested AgNO_3_ concentrations, 4 mg/L in the co-cultivation medium produced a 54% GUS expression and 43.68% explant regeneration rate from cotyledon explants (Figure 1D). Higher or lower concentrations of AgNO_3_ did not show significant results when compared with the 4 mg/L concentration, but they produced greater results than the control obtained in L-cysteine or ascorbic acid. There were 1.9-fold improvements in GUS expression and explant regeneration efficiencies.

### The Effects of L-Cysteine + Ascorbic Acid and L-Cysteine + AgNO_3_ on GUS Expression and Regeneration Efficiency

The combination of L-cysteine (800 mg/L) + ascorbic acid (40 mg/L) produced an 88% GUS expression and a 71.19% explant regeneration efficiency. Both factors were 3.1-fold greater than the control. For L-cysteine + AgNO_3_, the maximum GUS expression at 94% and explant regeneration efficiency at 76.66% were recorded, each being 3.3-fold greater than the control (Figure 1E).

### Regeneration of Transformed Chinese Cabbage Plants

**FIGURE 2:**
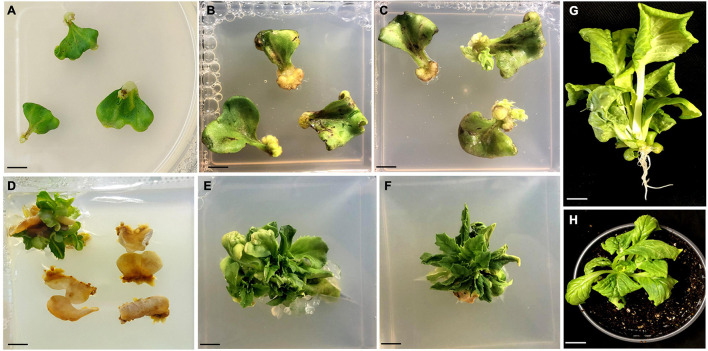
Regeneration of plants from GV3101 + pCAMBIA1303 infected cotyledon explants of Chinese cabbage. **(A)** Co-cultivated explants after a week. **(B)** Bulging of petiole region of the explants after 2 weeks **(C)** Initiation of small shoots from petiole region of the explants after 4 weeks. **(D)** Proliferation of shoots from cotyledon explants after 8 weeks. **(E,F)** Production of well-grown shoots from cotyledon explants after 11 weeks. **(G)** A rooted plant. **(H)** A hardened plant. All the cultures showed in **(A–F)** were grown in MS hormonal medium containing 800 mg/L L-cysteine, 4 mg/L AgNO_3_, 500 mg/L timentin, 12 mg/L hygromycin. Scale bar represents 1 cm.

The infected transformed cotyledon explants exhibited the ability to regenerate further into shoots, whereas the control or non-transformed explants never showed any regenerative responses and finally died upon selection (Figure 2). The escaped shoots were removed by three selection cycles, and the shoots were sub-cultured onto the new selection medium at 7-day intervals. The shoots were then proliferated and elongated (4–5 cm) in the same medium. Two shoots/explants were achieved in the same medium after 12 weeks of culturing (Table 1). The elongated shoots were then transferred to half-strength rooting medium containing 10 mg/L hygromycin B and 500 mg/L timentin for 5 weeks.

**TABLE 1:** Stable transformation efficiency of transgenic plants from cotyledon explants of *B. rapa*.

**Total no. of infected cotyledon explants**	**Total no. of shoots survived under third selection**	**Total no. of rooted shoots**	**Total no. of GUS positive plants**	**Total no. of Southern blot positive plants**	**Stable transformation efficiency (%)**
128	64.0^b^	38.0^b^	24.0^b^	18.0^a^	14.0^a^
128	72.0^a^	42.0^a^	26.0^a^	19.0^a^	14.8^a^
128	58.0^c^	34.0^c^	21.0^c^	17.0^a^	13.2^a^

*Values represent the mean of three experiments. Transformation efficiency = (number of southern positive plants/total number of infected cotyledon explants)/100. ^*a*−*c*^Means with the same letter are not significantly different at 5% level according to Duncan’s multiple range test.*

### GUS Expression Analysis

**FIGURE 3:**
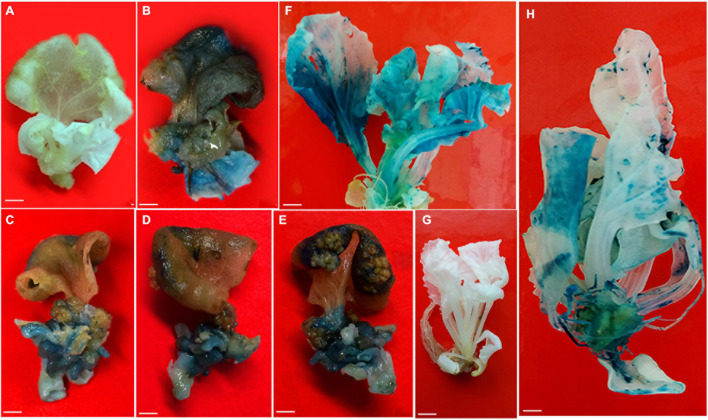
Stable expression of *gusA* gene in Chinese cabbage. **(A)** Control cotyledon explants with initiation of shoots from petiole region. **(B–E)** GUS expression in adaxial and abaxial sides of cotyledon explants with initiation of shoots. **(F)** GUS expression in fully grown shoots. **(G)** Control rooted plantlet. **(H)** GUS expression in rooted plantlet. Scale bar represents **(A–E)** 20 mm, **(F–H)** 1 cm.

The proximal end of the cotyledon explants stained blue owing to *gusA* gene expression. *Agrobacterium* infections might effectively have occurred at the proximal ends where the meristematic cells were actively involved in the differentiation followed by shoot bud formation. The GUS expression experiment revealed blue color formation in emerging shoots on explants, shoots and whole plants (Figure 3), whereas the non-transformed respective parts and plant (Figure 3) did not show blue color formation upon X-Gluc staining.

### PCR Confirmation and Copy Number Estimation by Southern Blotting

**FIGURE 4:**
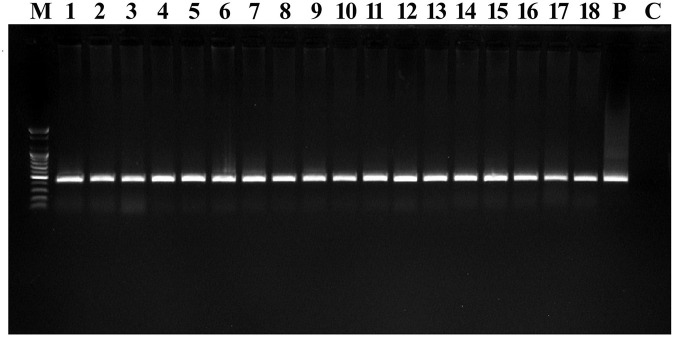
PCR confirmation in T_0_ transgenic Chinese cabbage plants. PCR amplification of 407-bp fragment of the *hptII* gene. Lane M 100-bp marker, lanes 1–18 DNA from transformed plants, lane P plasmid DNA (pCAMBIA1303; positive control), Lane C non-transformed plant DNA (negative control).

Amplified DNAs from transgenic plants exhibited bands near the expected 407 bp (Figure 4). This confirmed the presence of the transferred gene in the plant genome. The control did not show any amplification in the PCR analysis (Figure 4). To verify the number of copies and integration of *hptII* in the transgenic plants, Southern blotting was performed on control and transgenic plants (Supplementary Figure 1). DNA digestion of both control and transgenic plant samples was performed using *Eco*RI. The positions and numbers of bands for *hptII* appeared in different positions and represented different total copies in the transgenic plants (Supplementary Figure 1). The bands differed in individual transgenic plants, which necessitated autonomous random integration.

## Discussion

Setting up a high effective transformation is essential for the study of gene function and establishment of commercial transgenic lines in Chinese cabbage. Effective plant transformation systems involve the growth of transgenic shoots after infection with *Agrobacterium*. In the present study, we have tested different concentrations of hygromycin B to screen which concentration is lethal to Chinese cabbage shoot regeneration from cotyledon explants. Sivanandhan et al. (2016) reported that the selection agents in the medium play vital role in the development of transformed cells/shoots/roots after the explants were co-cultivated with *Agrobacterium*. Commonly, hygromycin B has been employed as an antibiotic in plant transformation systems. Most of the *Agrobacterium*-mediated transformation studies in Chinese cabbage reported either hygromycin or kanamycin as selection agents and very rarely mannose or bispyribac sodium (Ku et al., 2006; Min et al., 2007; Konagaya et al., 2013) to recover transgenic shoots. When different concentrations of hygromycin B were examined, 10 mg/L was selected as lethal concentration for the selection of transgenic shoots of Chinese cabbage. Most of the cotyledon explants was died when increasing the concentration of hygromycin B after 8 mg/L followed by 10 mg/L in selection medium. Jun et al. (1995) observed that regeneration ability of cotyledon explants was significantly decreased on kanamycin selection medium and the explants turned white within 7 days of culture in selection medium in *B. campestris* L. ssp. *pekinensis.* It has been reported that 25 mg/L kanamycin in the selection medium produced transgenic shoots of *B. campestris* L. ssp. *pekinensis* from cotyledon explants (Zhang et al., 2000). Cho et al. (2001) used 10 mg/L hygromycin B in *B. rapa* transformations. Lee et al. (2004) and Baskar et al. (2016) reported 10 mg/L hygromycin helped to screen the transgenic shoots from hypocotyl explants of Chinese cabbage (*B. rapa* L. ssp. *pekinensis*). However, Ku et al. (2006) and Min et al. (2007) reported 5 g/L and 7g/L mannose in the selection medium were suitable for Chinese cabbage (*B. rapa* L. ssp. *pekinensis*) and (*B. campestris* L. ssp. *pekinensis)* transformation using hypocotyl and cotyledon explants, respectively. Vanjildorj et al. (2009) reported that 10 mg/L hygromycin B was included in the medium used to select transgenic *B. rapa* plants. Konagaya et al. (2013) reported low escapes rate when 0.25 mg/L bispyribac sodium was added in the selection medium to select transformed shoots of (*B. rapa* L. ssp. *pekinensis*) using hypocotyl explants. Kanamycin at 17.17 μM in selection medium showed good recovery of transgenic shoots from leaf explants of Chinese cabbage (*B. rapa* L. ssp. *pekinensis*) (Liu et al., 2018). Li et al. (2021) used 25 mg/L hygromycin B to select transgenic plants from cotyledon explants of *B. rapa*.

Slightly increasing the concentration of selection agents from the minimum inhibitory concentration by passing one more cycle with a week interval has greatly contributed in recovery of true-to-type of transgenic plants (Sivanandhan et al., 2015). Hence, in order to increase recovery rate and minimize the escapes of transgenics, we applied three selection cycles with gradual increase (10, 11 and 12 mg/L) of hygromycin B concentrations to promote the selection of transgenic *B. rapa* explants. Jun et al. (1995) reported removal of kanamycin during subculture of transformed shoots after 4–5 weeks in *B. campestris* L. ssp. *pekinensis* transformation. Zhang et al. (2000) and Baskar et al. (2016) reported immediate transfer of co-cultivated explants to selection medium and constantly maintained 25 mg/L kanamycin and 10 mg/L hygromycin up to complete regeneration of transgenic plants in *B. campestris* L. ssp. *pekinensis* and *B. rapa* L. ssp. *pekinensis*, respectively. Similarly, Ku et al. (2006) and Min et al. (2007) reported direct transfer of co-cultivated explants to selection medium and constantly maintained 5 g/L and 7 g/L mannose up to complete regeneration of transgenic plants of Chinese cabbage (*B. rapa* L. ssp. *pekinensis*) and (*B. campestris* L. ssp. *pekinensis)*, respectively. Lee et al. (2004) grew transgenic shoots of Chinese cabbage (*B. rapa* L. ssp. *pekinensis*) after direct transfer of explants to selection medium that contained 10 mg/L hygromycin. Konagaya et al. (2013) excluded selection agent, bispyribac sodium for a week after co-cultivation and after elimination of *Agrobacterium* growth, the infected explants were transferred to selection medium and continuously maintained up to complete regeneration of transgenic shoots in *B. rapa* L. ssp. *pekinensis.* It has been reported that immediate transfer of co-cultivated explants into kanamycin and hygromycin B media without delay showed increasing recovery rate of transgenic plants in Chinese cabbage (*B. rapa* L. ssp. *pekinensis)* (Liu et al., 2018; Li et al., 2021). No other earlier studies involving three selection cycles with slight high concentration (steady increase of 1 mg/L) have reported in Chinese cabbage. To our knowledge, this is the first study that carried out with three selection cycles along with gradual increase of selection agent in the medium up to complete shoot regeneration in Chinese cabbage. The selection cycle strategy might be helped to recovery a good numbers of true-type transgenic plants as reported by Sivanandhan et al. (2015) in *W. somnifera*.

In *Agrobacterium*-mediated transformation, tissue browning and necrosis are common troubles in plants (Dan, 2008). In Chinese cabbage transformations, these are also main issues that lead to low levels of T-DNA delivery and competent cell regeneration from explants (Li et al., 2018). In the present study, tissue browning followed by tissue necrosis has been found in petiole region of cotyledon explants after three-day co-cultivation (Figure 1F), and further processing led to poor or no shoot regeneration. Different L-cysteine concentrations (0, 200, 400, 600, 800, 1000 mg/L) were added to the co-cultivation medium, and subsequently in the selection medium to prevent explant damage after infection with *Agrobacterium* for good regeneration of shoots. L-Cysteine at 800 mg/L in co-the cultivation medium resulted in higher GUS expression and shoot regeneration response from cotyledon explants. Earlier studies reported positive role of L-cysteine in *Agrobacterium*-mediated transformation in plants. For instance, addition of 400 mg/L L-cysteine resulted a high transformation efficiency in maize embryo (Frame et al., 2002). Olhoft et al. (2001) observed improvement of transformation efficiency in soybean when the cotyledon explant grew on 400 mg/L or 1000 mg/L L-cysteine medium. Sivanandhan et al. (2015) noted less browning of explants followed by increased rate of transformation efficiency in *W. somnifera* when the explants co-cultivated on 100 mg/L L-cysteine. Wound- and pathogen-defense related pathways are on in plant cells when the explants experience *Agrobacterium* or pathogen infection, wounding and environmental stresses. As a result of defense mechanism, secondary metabolites and phytoalexin work as a fungicidal or bactericidal or repellants to construct dead cell barrier from the existing adjacent plant cells in order to avoid the intrusion of foreign bodies into the plant cells (Heath, 2000). In this condition, L-cysteine acts as an antioxidant through the thiol group by inhibiting injury or pathogen-related protective reactions, and thereby restricts tissue necrosis and tissue browning owing to enzymatic reactions during *Agrobacterium*-mediated plant transformation when added to the co-cultivation medium (Olhoft et al., 2001). Liu et al. (2017) reported that transcriptomic data revealed oxidation reduction related genes, which were upregulated higher in the cysteine treated cultures in maize transformation. Many genes majorly related with oxidation reduction process or oxidoreductase activity process and metabolic process were identified in cysteine treated culture of maize in *Agrobacterium*-mediated transformation. The authors concluded in their study that cysteine may involve in relax the cell wall by changing cell wall and membrane metabolism when *Agrobacterium* infection occurs. It led to encourage the infection of *Agrobacterium.*

The abilities of various ascorbic acid concentrations to prevent damage to explants after infection with *Agrobacterium* were analyzed. The co-cultivation medium containing 40 mg/L ascorbic acid exhibited maximum GUS expression and percentage of explant regeneration levels. *Agrobacterium* infections induce reactive oxygen species (ROS) accumulation because of oxidative burst responses, and this process leads to plant cell death owing to necrosis (Bakshi et al., 2011). Over accumulations of ROS in the plant cells decrease the capability of *Agrobacterium* to infect plant cells and transfer T-DNAs (Dan, 2008). As a result, ascorbic acid, as a scavenging chemical, significantly enhanced GUS expression in, and shoot regeneration from, infected Chinese cabbage explants. Similarly, Bhatt et al. (2021) reported maximum transformation efficiency was recorded in the presence of 2.5 mg/L ascorbic acid along with L-cysteine and silver nitrate in the pretreatment solution of Kodo millet callus.

Silver nitrate as an inhibitor of ethylene played an important role in stimulating regeneration and morphogenesis of plants including Chinese cabbage (*B. rapa* L. ssp. *pekinensis*) (Sivanandhan et al., 2019). The present study confirmed that the inclusion of AgNO_3_ in the co-cultivation medium and subsequent selection medium slightly improved the survival capability levels of explants infected with *Agrobacterium*. The co-cultivation medium containing 4 mg/L AgNO_3_ exhibited higher GUS expression and percentage of explant regeneration levels under particular culture conditions. The ethylene rate was increased under stress condition, such as *Agrobacterium* infection with explants. This could lead to lethal effect in regenerating shoots. Silver nitrate in co-cultivation and selection medium significantly inhibited the ethylene production during regeneration of shoots after *Agrobacterium* infection (Mukhopadhyay et al., 1992). A higher transformation efficiency was obtained by adding AgNO_3_ to the co-cultivation and selection medium of *Brassica campestris* (Mukhopadhyay et al., 1992) and *Prunus avium* (Sgamma et al., 2015).

The optimum concentrations of L-cysteine + ascorbic acid and L-cysteine + AgNO_3_ were combined in the present study to try to achieve still greater GUS expression and percentage of explant regeneration levels. The combination of L-cysteine (800 mg/L) + AgNO_3_ (4 mg/L) produced maximum GUS expression and explant regeneration efficiencies, followed by L-cysteine + ascorbic acid. These results were greater than those recorded after using the optimum concentration of each compound independently. Generally, some physiological phenomena, like oxidative bursts with ROS formation, phenolization and successive cell death, occur during *Agrobacterium* infection/interaction with plants cells (Dan, 2008). The enzymatic browning and necrosis of explants during *Agrobacterium* transformation involves three action modes: (1). The phenomena occurred from within the infected explants; (2). Necrotic explants produced some antibacterial substances; and (3). The formations of some chemical signals for *Agrobacterium* induction dramatically block T-DNA transfer into the infected explants (Kuta and Tripathi, 2005; Dan, 2008). However, the enzymatic browning and necrosis may be overcome by adding antioxidative compounds to the media. Dan (2008) reviewed ascorbic acid and L-cysteine’s use as antioxidants to prevent enzymatic browning and necrosis which occur during *Agrobacterium* infection of explants. Thus, in the present study, we tested their effects, along with those of AgNO_3_, alone or in combinations on Chinese cabbage transformation. The combined effects of these compounds significantly increased the GUS expression level and the explant regeneration efficiency in Chinese cabbage. Enríquez-Obregón et al. (1999) found reductions in necrosis and hypersensitivity cell death rates (80%) of explants in rice transformants are cultured on L-cysteine + ascorbic acid + AgNO_3_ supplemented medium. The combination of these three compounds have a synergistic effect on the inhibition of hypersensitivity responses in the meristematic tissues of rice explants during transformation (Enríquez-Obregón et al., 1999).

The regeneration of transformed shoots from infected cotyledon explants was performed using optimized parameters (involving 800 mg/L L-cysteine, 4 mg/L AgNO_3_, 500 mg/L timentin and 12 mg/L hygromycin B in the media, a 10-min infection time, 3 days of co-cultivation, *Agrobacterium* of OD_600_ = 0.6 and 5 mg/L acetosyringone). The numbers of roots were less than those achieved in using the standard plant-tissue culture method (Sivanandhan et al., 2019) during the same culture period. During the entire process, the cotyledon explants passed through several stages, which hinder their *Agrobacterium*-mediated regeneration ability as observed by Karthikeyan et al. (2011) in rice transformations. The transformed regenerated plantlets were shifted to subsequent hardening, and a transformation efficiency of 14% was achieved (Table 1), which is significantly higher than earlier reports (Jun et al., 1995; Zhang et al., 2000; Lee et al., 2004; Ku et al., 2006; Min et al., 2007; Konagaya et al., 2013; Baskar et al., 2016; Liu et al., 2018; Li et al., 2021). Various transformation efficiencies (0.6-10.38%) in *B. rapa* L. ssp. *pekinensis* and *B. campestris* L. ssp. *pekinensis* were elaborated above based on Southern blot confirmation. However, Ku et al. (2006) and Baskar et al. (2016) reported transformation efficiencies of 83.3% and 15%, respectively, in *B. rapa* L. ssp. *pekinensis* by PCR confirmation. Hygromycin B-resistant and GUS-expressing plants, along with non-transformed plants, were selected randomly and subjected to a PCR analysis with *hptII* primers. PCR analysis confirmed the presence of *hptII* gene in the transgenic plants. In Southern blotting analysis, the transgenic Chinese cabbage plants exhibited the clear integration of the *hptII* gene in either single or multiple copies in their genomes (Supplementary Figure 1).

## Conclusion

A simple and reproducible *Agrobacterium*-mediated transformation method was developed to improve transformation efficiency for recalcitrant Chinese cabbage (*B. rapa* ssp. *pekinensis*) by adding antioxidants along with silver nitrate to the transformation media. A transformation efficiency of 14% was obtained in the present optimized protocol, which consisted 800 mg/L L-cysteine, 4 mg/L AgNO_3_, 500 mg/L timentin and 12 mg/L hygromycin B with three selection cycles in the media, 10 min infection time, 3 days co-cultivation, *Agrobacterium* growth to OD_600_ = 0.6 and 5 mg/L acetosyringone. This is the first report of optimizing components for Chinese cabbage *Agrobacterium*-mediated transformation, and this protocol may be useful in the significant improvement of Chinese cabbage traits through genome-editing tools, functional genomics, metabolic engineering and RNA interference studies.

## Data Availability Statement

The original contributions presented in the study are included in the article/Supplementary Material, further inquiries can be directed to the corresponding author.

## Author Contributions

GS performed most of the experiments and wrote the manuscript. JM, CS, SB, ZY, and SJ contributed to the data, visualization, formal analysis, and southern blotting and validation. SC supervised the experiment. S-GK reviewed the manuscript. YPL designed and led the experiment and acquired fund. All authors have read the manuscript and approved the submitted version.

## Conflict of Interest

The authors declare that the research was conducted in the absence of any commercial or financial relationships that could be construed as a potential conflict of interest.

## Publisher’s Note

All claims expressed in this article are solely those of the authors and do not necessarily represent those of their affiliated organizations, or those of the publisher, the editors and the reviewers. Any product that may be evaluated in this article, or claim that may be made by its manufacturer, is not guaranteed or endorsed by the publisher.

## Footnotes

1
https://www.naturalpedia.com

2
http://www.fao.org
