# Supplementary material for: L-Cysteine Increases the Transformation Efficiency of Chinese Cabbage (Brassica rapa ssp. pekinensis)
Source: Front Plant Sci. 2021 Oct 26;12:767140. doi: 10.3389/fpls.2021.767140 (PMC8576496; doi:10.3389/fpls.2021.767140)
Supplement: Supplementary file 1 [file Image_1.pdf]

Supplementary Material

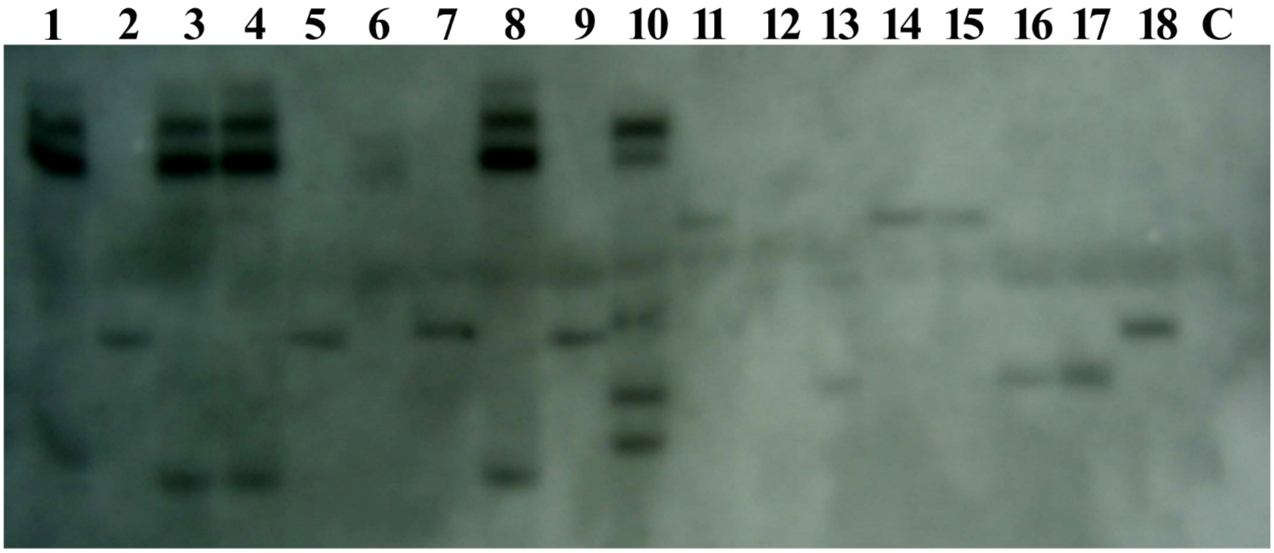

Supplementary Figure 1. Copy number estimation by Southern blotting in T_0 transgenic Chinese cabbage plants**.** Southern hybridization analysis performed to confirm the T-DNA integration in T_0 plants using DIG High Prime DNA Labelling and Detection Starter Kit II. Lane 1-18 DNA isolated from putative transformed plants, lane C non-transformed plant DNA (negative control).
